# Meditative Movement for Depression and Anxiety

**DOI:** 10.3389/fpsyt.2013.00071

**Published:** 2013-07-24

**Authors:** Peter Payne, Mardi A. Crane-Godreau

**Affiliations:** ^1^Microbiology and Immunology, Geisel School of Medicine at Dartmouth, Lebanon, NH, USA; ^2^Research and Development Service, Veteran’s Administration Medical Center, White River Junction, VT, USA

**Keywords:** Qigong, Chi Kung, Taijiquan, Tai Chi, exercise, basal ganglia, default mode network, interoception

## Abstract

This review focuses on Meditative Movement (MM) and its effects on anxiety, depression, and other affective states. MM is a term identifying forms of exercise that use movement in conjunction with meditative attention to body sensations, including proprioception, interoception, and kinesthesis. MM includes the traditional Chinese methods of Qigong (Chi Kung) and Taijiquan (Tai Chi), some forms of Yoga, and other Asian practices, as well as Western Somatic practices; however this review focuses primarily on Qigong and Taijiquan. We clarify the differences between MM and conventional exercise, present descriptions of several of the key methodologies of MM, and suggest how research into these practices may be approached in a systematic way. We also present evidence for possible mechanisms of the effects of MM on affective states, including the roles of posture, rhythm, coherent breathing, and the involvement of specific cortical and subcortical structures. We survey research outcomes summarized in reviews published since 2007. Results suggest that MM may be at least as effective as conventional exercise or other interventions in ameliorating anxiety and depression; however, study quality is generally poor and there are many confounding factors. This makes it difficult to draw definitive conclusions at this time. We suggest, however, that more research is warranted, and we offer specific suggestions for ensuring high-quality and productive future studies.

## Introduction

While both exercise and meditation have been acknowledged as having health benefits, the category of exercise that combines meditative focus with movement is often ignored or misunderstood. As a class of exercise it lacks a broadly accepted name. We refer to the group of practices including the traditional Chinese practices of Taijiquan (1), Qigong (2), and Hatha (postural) Yoga (3), as well as Western methods such as the Alexander Technique (4) and Feldenkrais (5). It has been proposed that this form of exercise be called “Meditative Movement” (MM), which is defined by Larkey et al. as a practice involving movement, a meditative state of mind, attention to the breath, and deep relaxation (6). We will use the designation MM except when referring to a specific discipline.

We address MM because of the proliferation of publications, some scholarly, some in the popular press, that claim physical or psychological benefit from one or more of these practices, including reduced anxiety and depression, a more positive affective state, greater calmness of mind, greater physical relaxation, improved general health, better balance, lower blood pressure (BP), and improved biomarkers for inflammation and immune function [such as C-reactive protein (CRP) and cortisol] (7–13).

Conventional exercise has been shown to improve depression and anxiety (14, 15), but it is not clear whether these results also apply to MM, or whether the mechanisms of MM are similar or different to those of conventional exercise. Likewise there is a substantial body of literature on (seated) meditation; it is likely MM shares many of the same mechanisms as meditation, but they are distinct practices. The objective of this review is to define MM and to clarify how it differs from conventional exercise and from seated meditation, to examine the evidence for its ability to ameliorate depression and anxiety, and to suggest possible mechanisms for these effects.

## Methods

To gather preliminary information for the survey of the current state of research into the effects of MM on anxiety and depression, we performed searches in PubMed, Ovid, and Google Scholar, using the key words Qigong, Taijiquan, Tai Chi, Chi Kung, exercise, Yoga, mindful, movement, meditative, meditation, Somatics, Alexander Technique, and Feldenkrais, combining these terms with psychological, health, psychosocial, stress, well-being, depression, and anxiety. We confined our search to publications in English, and prioritized reviews later than 2006. We found 14 review papers focusing solely or substantially on MM in relation to anxiety or depression (16–29), all of them relating to meditation, Yoga, Qigong, and/or Taijiquan, and used these as our principal source of information. In some cases we included general reviews of MM as long as they included a substantial focus on affective states (18, 19, 29).

For the purpose of this study we excluded Yoga, as well as studies focusing solely on seated meditation. Due to the many systems covered by the word “Yoga” (explained in, see Yoga below) we chose to substantially limit our use of studies of Yoga. Future studies of MM could include Yoga as long as care is taken to separate the more religious or philosophical aspects from the MM components and to examine carefully the specific techniques to determine whether they meet the definition of MM. We also chose not to explore the literature on seated meditation in detail, except in specific cases where it directly illuminates MM, for the reasons explained in Section “Seated Meditation.” Some academic reviews of MM practices have, however, included seated meditation (16, 30). The traditional Chinese practices of Taijiquan and Qigong conform well to the proposed definition of MM (6), therefore our study focuses principally on them.

We found no relevant reviews of Somatics, Feldenkrais, or Alexander Technique. For further information on aspects of MM not mentioned in the scientific literature, we relied on the authors’ extensive libraries of books and articles on the subject, supplemented when necessary by searches on Amazon.com and the Library of Congress.

Limitations of these methods were that we did not include reviews in languages other than English; however, because several of the reviews we included did not have this limitation, we nevertheless were able to take into account much of the work published in China and Korea. We were however unable to access directly research papers in languages other than English, which is a significant limitation of this study. Also, we focused mainly on Qigong and Taijiquan, excluding the literature on Yoga and much of the literature on meditation for the reasons explained above. A deeper investigation of the specific nature of the techniques of Yoga and meditation studied in specific papers might allow a more inclusive approach in the future, and might lead to changes in the definition of MM. In addition we chose not to search for every possible kind of practice that might be a form of MM. Some initial exploration found no relevant references in the scientific literature to several names of Somatics or Asian MM systems (for example, Alexander Technique, Feldenkrais, Baguazhang), and we concluded that continuing this search would be unfruitful and would take too much time, besides necessarily being incomplete as the authors are not familiar with all forms of practice that might be considered MM. Nevertheless, this is an acknowledged limitation of this study. A more thorough search using more terms might well discover more information of value.

## Defining the Terms

“Qigong” and “Taijiquan” often appear in Western literature with multiple spellings. There are two systems of transliteration, the earlier Wade–Giles system, and the contemporary (and official) Pinyin. By way of introducing these alternative spellings, we will use the Pinyin form, with the Wade–Giles and other alternate spellings in parentheses, and a phonetic approximation in italics in our definitions below.

### Qigong

“Qi” (Ch’i, *chee*) means “life energy” or “breath.” It is a core concept throughout Chinese culture. “Qigong” (Ch’i Kung, Chi Kung, *chee goong*) means “work on the life energy,” and is a broad term including methods that simultaneously “regulate the body, the breath, and the mind” (1). There are four main branches of Qigong: health-maintenance Qigong, medical Qigong, martial Qigong, and spiritual Qigong; all share the same basics, but use different techniques relevant to their specific aims.

### Taijiquan

Taijiquan (T’ai Chi Ch’uan, Tai Chi Chuan, Tai Chi, *tie jee chwahn*) is a Chinese martial art based on the same principles as Qigong; basic or modified Taijiquan is also used as a health and meditative exercise. Opinions differ as to whether Taijiquan should be regarded as a form of Qigong or as a separate category (16, 30); here, we consider both Qigong and Taijiquan as forms of MM.

### Seated meditation

Seated meditation uses a mostly static posture, and involves the exploration of various mental states. Many different form of seated meditation are practiced throughout the world. There is substantial overlap between the techniques of MM and some forms of seated meditation, in that they may use similar mental strategies. However the term MM implies that movement or the intention of movement is involved (6), which is not true of many forms of meditation. There is a large literature on seated meditation, especially the style called “mindfulness,” and comparatively little on MM *per se*.

### Yoga

The Sanskrit word “Yoga” means “to join,” and refers to the individual’s union with God; traditionally, Yoga is the practical side of the diverse Hindu religious systems. Different forms of Yoga use differing methods to achieve union with God. Some forms of Yoga may be forms of MM as defined in Section “Characteristics of MM” below, such as Hatha Yoga asanas and pranayama (3) and Yantra Yoga (31); others, such as Bhakti, Jnana, and Raja Yoga (32), are specialized forms of devotional practice, philosophical inquiry, or sitting meditation, and often involve moral and dietary observances.

### Other Asian systems

Meditative movement includes more Asian systems than those mentioned above; a full enumeration would not be useful here as the numbers run into the hundreds, but Aikido (33), Shin Tai Do (34), Baguazhang (35), Sufi Dance (36), and Buddhist walking meditation (37) are other exemplars.

### Somatics

Over the past century, a number of MM practices have developed in the West. These have been referred to by the name “Somatics” (38). This term includes a wide variety of practices that, in general, share the defining characteristics of MM; in addition to the Alexander Technique (4) and Feldenkrais (5), examples of Somatic practices are: Eutony (39), Mensendieck (40), Focusing (41), Sensory Awareness (42), Aston Patterning (43), Rolfing Movement (44), Continuum (45), and Authentic Movement (46). Somatic Experiencing^®^ (47) uses the principles of MM as a therapy for trauma. At this point there are very few scientific studies of these methods, and we found none focusing on affective states.

## Why “Meditative Movement?”

In the past 5 years, the number of research studies of MM has increased considerably (47); however, these practices have often been treated by researchers as forms of exercise comparable to ordinary aerobics, stretching, or relaxation techniques. As Catherine Kerr pointed out (48), the understanding of the biomedical researcher as to what is happening in MM is often at variance with that of the MM teacher. A researcher might understand the MM practices as a form of exercise for generalized stress reduction, not much different in principle from going for a walk. The MM instructor, however, thinks of it as a more sophisticated process, in which the awareness of the practitioner is placed in specific regions of the body to make specific changes. This disjunction of views can lead to problems in designing experiments that ask and answer relevant scientific questions (49).

The use of the term “MM,” suggested by Larkey (6), is an important step in recognizing the special features of these practices. Larkey proposed the following essential characteristics of MM: first, a meditative state of mind, usually involving a focus of awareness on the body; second, some form of prescribed (or sometimes spontaneous) movement; third, explicit attention to the breathing; and fourth, a state of deep relaxation. Larkey based his definition on his familiarity with Qigong, Taijiquan, and similar practices, in an attempt to bring attention to these forms of exercise as distinct from conventional exercise. We will expand somewhat on these in order to define more precisely the features of MM, and to make it clear how radically it differs from most other exercise. We believe that, for future research, it is important to have a full and precise understanding of MM on its own terms, so that it can be accurately evaluated.

## Characteristics of MM

### Mind

“Mind” in this context means “awareness” and not “conceptual thought.” In MM the mind is neither engaged in conceptual activity nor focused on a future goal, nor is it in the “default mode” of mind-wandering (50), but instead is focused on direct bodily experience. We view this spatial/interoceptive/proprioceptive/kinesthetic focus of awareness as the principal defining characteristic of MM. The practitioner’s awareness is on the kinesthetic sensations of the whole body moving through space; the flow of breath and blood and other visceral sensations; the experience of balance, orientation, and posture; and the felt sense of space – quite different from physical awareness in conventional exercise (51). Kerr (49) has referred to this focus as “mind-in-body,” to distinguish it from the more familiar concept of “mind-body” practices. This way of using the attention is similar to that used in some forms of seated meditation (52); but MM often involves additional specific mental techniques. For example, practitioners may be instructed to “feel the air as heavy” (53), as if they were moving underwater. This may be difficult at first, but with practice one can develop a vivid tactile/kinesthetic sense of viscous, almost hydraulic air movement, accompanied by a pleasurable sense of lightness, warmth, smoothness, and power (53). This indicates progress in the exercise and may be predictive of positive objective effects. In MM practices, the mind is also used to “direct the movement of Qi” (54). Since this is a core aim of most MM, it is important to understand this in a way that is compatible with a scientific approach. Section “Imagery” below explores this in more detail.

### Movement

Meditative movement may use either prescribed movement (where the required motion is specific and must be learned and practiced) or spontaneous free-form movement (where the practitioner allows their body to move spontaneously on its own). In some cases, the movement used may be extremely subtle, to the point of being invisible (55). In order to move the Qi (cause certain sensations in the body), physical movement is useful but not necessary. A practitioner may begin by making a large and obvious motion, then make it smaller and smaller until it is imperceptible. In this process the interoceptive/proprioceptive sensations become progressively more intense. This increased intensity may correlate with the objective effectiveness of the practice. A common saying in Qigong is, “Small movement is better than large movement; no movement is better than small movement” (56). In traditional Qigong practice, quiescent seated meditation is considered to be a part of Qigong, as is quiet standing. Although the distinction between “static” and “moving” practice is acknowledged, it is not a major distinction; in traditional Qigong, they were usually practiced together, with each supporting the other (57). In many apparently static forms of Qigong, the mind is actively employed in imagining movement, which produces certain internal sensations of motion; such a practice may alternate between overt and imperceptible “movement.” In this context, it becomes hard to define exactly what is meant by the distinction between movement and stillness. We wonder whether, logical as it seems at first, making overt movement an essential element in MM is appropriate. One possibility would be to refine this part of the definition of MM to refer to the intention of movement, or to include the movement of internal sensations in the body in the definition of “movement.”

### Breathing

Awareness and control of the breath are central in Qigong and Taijiquan (58), Hatha Yoga (59), and Somatics (8). In Chinese and in many other languages, the same word can refer to both “breath” and “life energy.” “Attending to or moving the Qi” can refer either to the physical breathing, or to certain bodily, emotional, or spatial sensations (58). These multi-level meanings of key terms must not be ignored in approaching MM. Altering the breathing pattern may alter the functioning of the autonomic nervous system (60). Various breathing practices are said to enable emotional release (61), to calm the mind (62), or to enhance physical power (63). A central practice in most forms of Qigong is to pass the awareness through the body in synchrony with the breathing rhythm (64). Depending on the desired result, the breath may be slow or fast, felt in various parts of the body, imagined as having different qualities (such as warmth or coolness), or held for various lengths of time (65). In MM, the breath is described as a bridge between unconscious and conscious functions, a way for the conscious mind to influence the unconsciously controlled functions of the autonomic nervous system (66). The mutual influence of the breath, the autonomic nervous system, and the emotions is well recognized in the scientific literature (60, 67–76), and numerous studies have been made of the effects of specific MM breathing techniques (77–83), but as far as the present authors are aware there has been no systematic review of these studies.

### Deep state of relaxation (balanced tone)

In English “deep relaxation” conjures images of a floppy slackness. On the other hand, we can talk of a jaguar walking through the jungle as “deeply relaxed”; these are two different states. The Chinese word usually translated “relax” is “song” (*soong*) 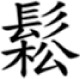 (84). However, this word does not mean limpness (84), but rather a state of completely balanced tone, “eutonis” (39, 85), in which every muscle is doing exactly what it should. This state is experienced as light, free, open, and effortless; but at the same time stable, powerful, and well-rooted. Tension is a state of hypertonus, slackness a state of hypotonus, and the outcome of successful MM practice is a state of eutonis. There are practices that involve a hypotonic state, like Hatha Yoga’s Shavasana, or “corpse posture” (3); some practices use brief maximal hypertonus. Neither of these is typical of MM, which always aims at the balanced state described above.

Biological systems are “complex systems”; this is a technical term implying spontaneous self-organization (86, 87). Complex systems organize themselves to preserve optimal, rather than minimal or maximal, levels of any number of variables (such as temperature or chemical composition). This is known as “homeostasis,” a term coined by Cannon (88). Cortisol levels, for instance, can be an indicator of stress, a presumptively negative condition; too little cortisol, however, is as bad as too much (89, 90). Great care is needed in pharmacological interventions to avoid unwanted side effects due to “overshoot.” The aim of MM is to restore the body’s innate mechanisms for establishing homeostasis or dynamic equilibrium (58). Keeping this simple point in mind could change a research hypothesis from “does this intervention reduce cortisol levels?” to “does this intervention move cortisol levels toward a normal range?”

A complex system that is poorly self-regulated may fail to maintain a variable (such as the level of hormones or other signaling agents) within a normal range; or the level of concentration may oscillate over time, between too low and too high. Increased self-regulation will reduce the magnitude of oscillations so they no longer leave the normal range; more refined regulation results in smaller oscillations (91). Investigations of body sway suggest that MM may also improve postural self-regulation (92).

In studying MM it should be kept in mind that the desired outcome is a state of increasingly refined dynamic balance, and not a state characterized by maxima or minima, tension, or slackness. The word “relaxation” should be used with caution due to its ambiguity, and the concepts from the theory of complex systems may prove useful in describing the outcomes and processes of MM.

## Research

### Benefits of MM

Meditative movement has positive effects on a wide range of mental and physical measures, although results are far from unambiguous. There have been a number of publications on the effects of MM practices on: depression (23), anxiety (17), cognitive ability (93), inflammation (94), immune function (95), arthritis (96), supportive cancer care (97), cardiac and pulmonary health (78, 98, 99), balance (98, 100), aerobic capacity (101), strength (102), bone density (103), fibromyalgia (104), and diabetes (105). Overall, MM seems to have positive effects on a broad range of health conditions. An equally consistent finding is that the vast majority of studies have serious limitations, and much more high quality research is needed to be able to draw definitive conclusions (11, 19, 20, 48).

### Reviews of MM’s effects on affective states

A number of reviews have looked specifically at the effects of Taijiquan, Qigong, Yoga, and seated meditation on improving anxiety, depression, and other affective measures (16–29). A relatively small number of studies have focused on the use of MM for improving anxiety and depression, whereas a larger number of studies have investigated the effects of seated meditation (106, 107). Here we focus principally on MM rather than seated meditation.

Ospina (29) reviewed the scientific quality of studies of meditation, defined as including Yoga, Taijiquan, and Qigong as well as seated meditation (30), and, applying the rigorous CONSORT standards (108, 109), concluded that most studies were of poor quality. Further scientific research into meditation required better attention to study quality as well as a more unified theoretical perspective on meditation. She stated that no firm conclusions about the effects of meditation could be drawn from the available research (30).

A more recent review and meta-analysis by Chen at al. focusing on MM for anxiety (16), examined 36 adequate randomized controlled trials (RCTs). This study used the classical Chinese definition of Qigong as “the skill of mind-body exercises that integrate body, breath, and mind adjustments into one” (16), and thus included sitting meditation practices. In evaluating studies for inclusion, Chen advocated the use of the Boutron modification (110) of the rigorous CONSORT standards. The CONSORT standards (108, 109) were designed to apply to the evaluation of design quality of pharmaceutical trials, where rigorous double-blinding is easy. In studying MM however, the same level of blinding is more problematic; the Boutron modification gives guidelines specifically for non-pharmaceutical studies.

Twenty-five of the 36 RCTs investigated by Chen (16) found meditation (seated or moving) significantly more effective than control interventions in improving the symptoms of anxiety. Control groups included standard of care only (no intervention), attention controls (using another activity unrelated to MM), and active interventions other than MM (16). Comparing Standard Mean Difference (SMD), there were two statistically significant differences between different groups of studies: outcomes of poorer quality studies tended to be more positive, and studies from Asian countries also tended to more positive outcomes. There were also indications (although not statistically significant) that movement-oriented methods were more effective than static meditation, and that group delivery was better than individual. Most studies did not focus on clinically diagnosed anxiety disorders, but used anxiety questionnaires as one among several measures, leaving it unclear how effective these interventions would be for clinically significant levels of anxiety. No adverse effects were reported in any of the studies (16).

Jahnke selected 67 RCTs for review (out of 576 considered) (19). His criteria were that the study appeared in a peer-reviewed English-language journal between 1993 and 12/2007; that it had been cited in the academic literature; and that it was designed to test the effects of Taijiquan or Qigong (exclusive of seated meditation). Twenty-seven of the 67 studies focused on psychological outcomes, and 6 on immune and inflammatory outcomes. In most cases MM demonstrated improvement in measures of anxiety and depression; these changes were generally significant compared with inactive controls, but did not usually reach significance when compared to an exercise or other active therapeutic control. In six other studies, MM reduced stress markers such as cortisol, adrenaline, and noradrenaline, and inflammation markers such as cytokines, CRP, and immunoglobulin-G (Ig-G). These results gave a biochemical confirmation that MM may reduce the secretion of stress-related and inflammation-related biomarkers. Jahnke noted that MM rarely produced less change than active therapeutic controls, which was significant in view of the simplicity, safety, cost-effectiveness, and gentle exertion of MM practice. Jahnke’s conclusion was that “this category shows promise for examining (MM’s) potential mechanisms of action for the change of psychological states.” Jahnke made a case for considering Taijiquan and Qigong as substantially identical: his study revealed no significant differences in outcomes between Taijiquan and Qigong over a wide range of variables.

A review by Oh (21) on Qigong and depression found 10 acceptable RCT studies between 2009 and 2011. Oh excluded Taijiquan and meditation, including only moving Qigong practices. Only two of the studies focused specifically on depression as a primary outcome; the rest used a measure of depression as one among others. Of the 10 studies, 4 found that Qigong had significant positive effects on depression; 2 found that Qigong had the same positive effectiveness as conventional exercise, and 1 found that Qigong was as effective as conventional rehabilitation treatment. This latter study by Tsang (23) involved both a pilot study and a follow-up with a larger sample size, comparing Qigong to a specific rehabilitation program targeted at depression. Qigong had significantly better results than the rehabilitation group. Oh’s evaluation was that these results were inconclusive: he cited widespread problems with poor experimental design, lack of specificity in description of the intervention, small sample size, brief length of study, lack of investigation of biomarkers, lack of studies involving clinical cases, lack of three-arm studies, and inadequate blinding. While many studies indicate that Qigong may effectively reduce stress, the litany of experimental short-comings outlined above highlights the need for greater rigor in the design of future studies.

A 2013 review of the effects of Qigong on anxiety, depression, and well-being by Wang et al. (28) drew from both Chinese and English-language studies, since 2000 for the former and 2003 for the latter. One hundred and fifty-eight studies were identified, of which 15 were finally selected for inclusion. This review excluded Yoga and seated meditation, focusing exclusively on standard Qigong exercises involving obvious movement, and applied rigorous standards for study selection. In all but one study, subjects were healthy or had a non-psychiatric chronic illness; one study involved depressed patients. In seven of the studies, mood and depression scores improved significantly (using a variety of scales such as the Hamilton Depression Severity Index, the Self-Rating Depression Scale, and the CES Depression Scale). Compared to usual care, psychosocial support, and active stretching, Qigong did better in two studies, while in two studies Qigong did as well as the active control. Anxiety scores also decreased significantly in seven studies, two of these compared to an active control. One study looked at stress-related biomarkers (cortisol, catecholamines) and found significant reductions compared to a wait-list control. Quality of life (a general measure of well-being based on a questionnaire) and self-efficacy (a measure of how capable and confident the subject feels in relation to daily demands) also showed significant improvement, both compared to wait-list controls and to a traditional rehabilitation group (one study); in another study, Qigong trended toward being more effective than conventional exercise but not significantly so. A meta-analysis of three studies of diabetic patients demonstrated that Qigong was effective at reducing depression and anxiety and improving quality of life. The authors comment on the poor quality of most studies and the need for more careful study design, larger samples, and better controls. They suggested using a convincing “sham” Qigong control to reduce “frustrebo” effects (the possible negative effect on controls from not receiving the intervention they want).

The use of sham MM controls is rare in MM studies, and designing such a study presents challenges (111), however a RCT by Lee et al. (9) used an effective sham Qigong control. They found significant reduction of anxiety (as measured by Spielberg’s state-trait anxiety inventory, STA1-X1), as well as cortisol and aldosterone levels, in a Qigong (“Qi-training”) group as compared to sham Qigong controls. The control group learned exactly the same movements, but participants were not instructed how “to gather and move the Qi” (the process of intentional manipulation of interoceptive sensation). Lee states that after the 1-h training, the intervention subjects “could gather and move Qi with conscious effort” and that “[t]he control group learned to perform the same external motions as the Qigong training group, but without any conscious effort to gather or move Qi.” Measures were taken shortly before and shortly after each training session; sessions lasted 1 h, and were repeated three times, separated by at least 2 days. Care was taken to blind the randomization as well as the collection of outcome data. This study is one of the few that give adequate details about the intervention, including the subtle factors alluded to above, as well as providing a valid double blind in the assessment of outcomes. The results suggest a contribution to anxiety reduction by factors specific to MM (“gathering and moving the Qi”).

In a 2010 review focusing on Taijiquan, Fields (18) mentioned three studies showing the positive effects of Taijiquan on the autonomic nervous system as measured by heart rate (HR) variability frequency analysis; in two of these studies, Taijiquan was shown to have better results than brisk walking or slow movement, which has interesting implications for the comparison between conventional exercise and MM. Fields also mentioned four studies in which Taijiquan has been shown to decrease a variety of negative emotional states, as well as two of her own studies combining Taijiquan and Yoga practice for pregnant women, in which significant alteration in EEG measurements was shown. Like all the other reviewers, she pointed out the many methodological flaws in these studies, including a wide range of dosages, target populations, diagnostic instruments, and the frequent use of pre-post measurements rather than controls. A limitation of her review is that she does not give details of how she selected the studies to include.

Another 2010 review by Saeed (22) looked at conventional exercise and Yoga as well as Taijiquan, Qigong, and meditation. Saeed focused on studies of clinically diagnosed affective disorders. Although Yoga is not within the scope of the present study, it is interesting to note that there appear to be a number of studies of the effects of Yoga on clinical anxiety, depression, and other psychiatric diseases, in contrast to the literature on Qigong and Taijiquan where there are very few such studies. Saeed’s conclusion was that there was not enough evidence to support using Qigong or Taijiquan as a treatment for clinical affective disorders. Saeed’s survey excluded most of the studies included in other reviews, which measured changes in anxiety, depression, and mood among non-clinical populations. Saeed also found little evidence for the effectiveness of conventional exercise in these populations.

Yet another review in 2010 by Wang et al. (26) searched English and Chinese databases through 2009 for references to Taijiquan (not Qigong) in relation to anxiety, depression, mood, self-esteem, and psychological stress. Randomized, non-randomized, and observational studies were considered. Randomized controlled studies were assessed for inclusion using the Jadad criteria (112). Wang performed a meta-analysis (using a bias-corrected Hedges g-score to compensate for small sample size), and discussed the non-randomized studies, separately for each of the five affective measures (anxiety, depression, mood, self-esteem, and psychological stress). Overall results included significant support for the positive effect of Taijiquan on all five measures. He noted that, despite the use of exercise control groups in several studies, it was not possible to say whether Taijiquan had equal or superior effects compared to conventional exercise. He also noted the limitations in conducting a meta-analysis due to heterogeneity of population and dosage. The present authors note that very few of the studies tested the effects of daily practice of Taijiquan; most studies involved practice two or three times a week, which is generally regarded among Taijiquan teachers as inadequate for achieving significant results (113).

A recent 2013 review by Wang et al. (27) looked at Qigong (not Taijiquan). An extensive search of English and Chinese databases was done, inclusive of Chinese dissertations and doctoral theses. The selection of studies for inclusion in the review used narrowly focused criteria: only RCTs were included; subjects had to have a diagnosis of anxiety or depressive illness; studies of mood, self-esteem, or stress were not included; studies had to use instruments validated for the measurement of depressive or anxiety states. The Wayne checklist (103) was used to evaluate study quality; this is a checklist developed specifically for use in studies of Qigong. It includes several items clearly required by the nature of the Qigong intervention, such as a complete description of the intervention and details of the qualifications of the instructors, as well as appropriate blinding requirements. Following data extraction, the effect sizes were calculated using Hedges g-score, and SMD was calculated for pooled effects. Out of 503 studies, 12 RCTs met inclusion criteria. Three of these used conventional exercise as a control. All studies measured depressive symptoms, whereas only four evaluated anxiety. Pooled effects were calculated for each type of control group used. Compared with conventional exercise, Qigong showed a moderate positive effect on measures of depression; compared with Cognitive-Behavioral Therapy, Qigong was equal in effect; compared with a newspaper reading control, Qigong showed a large effect; and compared with waitlist, a moderate effect. Among the four studies investigating anxiety, only one showed Qigong superior to controls. Wang cautioned that home practice was not monitored, thus dosage is hard to ascertain. Also, the Qigong intervention was always provided in a group context whereas the control groups often did not have this social exposure. Since social engagement may have a significant effect on depressive symptoms it is necessary in future studies to provide equal social engagement for all groups. Wang’s overall conclusion is that evidence for the effectiveness of Qigong in treating anxiety and depression is positive but limited by numerous factors.

A speculative 2008 review by Tsang (25) moved forwards from his earlier studies on the effects of Qigong on depression in the elderly (114, 115) to reflect on the possible neurobiological basis for these effects. His earlier studies investigated the psychological basis for improved depression. Qigong practice increased the sense of self-efficacy and mastery by enabling elders to master a physical task as well as to improve competence through increased balance and strength; in addition Qigong practice provided them with increased social support and improved interpersonal relationships. These psychological factors may cause decreased depression (116). Tsang offered three possible neurobiological explanations for Qigong’s anti-depressive effect. First, he suggested that Qigong may act by increasing brain levels of monoamine neurotransmitters, as has been claimed for selective serotonin reuptake inhibitors (SSRIs). He noted that studies have shown that conventional exercise has this effect (117), as well as increasing the concentration of tryptophan in the brain (tryptophan is a serotonin precursor). He pointed out that the time course of Qigong’s effect is similar to that of SSRIs: the antidepressant effect takes a couple of weeks to initiate, then fades away a few weeks after ceasing the intervention. Tsang’s second explanation involved the hypothalamic-pituitary-adrenal (HPA) axis which secretes cortisol and adrenaline in response to stress, and which has been implicated in depression. Tsang pointed out that, since the hypothalamus is under control of the limbic system, the mental calming effects of Qigong may reduce limbic activation of the HPA axis, thus reducing plasma cortisol and ACTH and possible lessening depression. Tsang cited studies showing that Qigong practice may reduce cortisol and ACTH (9). Tsang’s third explanation invoked the relation between decreased neurogenesis in the hippocampus and depression. Stress and the resultant increase in plasma cortisol may downregulate brain derived neurogenesis factor (BDNF) and reduce neurogenesis. He noted that animal studies suggest that exercise upregulates BDNF and increases neurogenesis (118), and that conventional exercise in humans may have the same effects (119, 120).

Many reviews of MM have noted its effects on biomarkers of inflammation such as CRP, tumor necrosis factor alpha (TNF-α), and interleukin 6 (IL-6) (19, 62, 93, 94). As Tsang pointed out, this is potentially relevant to some of the effects of MM on depression, as it appears that depression is linked to inflammatory processes in the brain (in particular the hippocampus), which interfere with the generation or survival of new neurons in the hippocampus (neurogenesis). In a recent review of the connection between depression and inflammation, Krishnadas pointed out that a third of patients with major depressive disorder (MDD), without any other major illness, have elevated inflammatory biomarkers; in particular, CRP, TNF-α, and IL-6. Moreover, MDD is epidemiologically associated with inflammatory medical conditions (121), and patients treated with cytokines are at increased risk of developing MDD. Neuroinflammation is linked with a number of psychiatric and neurological disorders in addition to depression (122). Since peripheral inflammation and central nervous system (CNS) inflammation involve somewhat different conditions, it is not possible to conclude from blood and salivary markers that MM might affect CNS inflammation. However melatonin may protect the brain against inflammation and promote neurogenesis (123), and meditation produced both short-term and long-term increases in melatonin levels (80, 124, 125).

### Conclusion

Overall, these reviews invite the following tentative conclusions: MM consistently produced reductions in measures of anxiety and depression when compared with non-active controls in studies of anxiety or depression. When matched to an exercise or other active therapeutic intervention control, MM usually performed about the same, rarely any worse, and sometimes better than the control. MM generally produced less reduction in measures of anxiety and depression in seriously ill patients and more reduction in healthy or slightly compromised subjects. However, the forms of MM used in such studies are invariably health-maintenance Qigong, and not the much more specifically targeted medical Qigong. It would be surprising if an intervention designed for mild ill health were to be effective for severe clinical conditions.

Review papers usually investigate multiple studies of pharmaceuticals or other well-defined treatments. Since the administration of a specific dosage of a particular drug is easy to standardize, variation of results between different studies is assumed to reflect random or uncontrolled factors not related to the drug itself; therefore a statistical averaging of many studies is assumed to give more accurate results as to the true effectiveness of the drug. However, the situation with MM is quite different. Given the complete lack of standardization or taxonomy of MM, divergent results in studies of MM may reflect in part the differential effectiveness of the interventions used rather than uncontrolled factors. For this reason, although we agree with several of the reviewers that the results are inconclusive, we feel that the remarkably positive results obtained in some studies, even when MM was compared to an active targeted intervention (23), warrant continued exploration of MM along the scientifically more rigorous lines we suggest below.

### Shortcomings of studies of MM

All the reviewers mentioned above (16, 18, 19, 21, 22, 25–29) come to similar conclusions about the shortcomings of most of the studies of MM to date. These short-comings fall into two broad categories.

The first category has to do with general experimental design (16, 19, 28, 126). This includes first, disagreement about the necessity of certain aspects of experimental design, such as blinding of the participants; and second, the clearly necessary and often inadequate aspects of the studies, such as small sample size, inadequate description of procedures, inadequate blinding of data collection, and inadequate controls. The former issue involves the nature of the MM intervention itself. Because an important part of most MM practices is the cognitive/affective state of positive belief and expectation, controlling for this variable could be seen as inappropriate, irrelevant, or unnecessary (111). Further complicating experimental design, in MM the placebo effect is not necessarily regarded as a confounding factor; rather, the factors on which the placebo effect is based are being harnessed to produce positive outcomes (111). It is possible to tease out the cognitive, affective, and motor aspects components of the effects of MM by good experimental design; however, MM might achieve some of its effects through the interaction of its component factors, rather than a simple addition.

The second category involves difficulties in experimental design specific to the study of MM (21, 49, 127). The study of MM presents issues such as difficulties with blinding and the selection of appropriate sham control activities. Studies present a wide range of interventions, often poorly specified; they use diverse doses and durations; and there is usually a mixture of possible active factors in a single intervention (such as expectation, psychosocial support, skill levels, training methodology, movement, mental focus, belief, and expectation). Much of this confusion stems from the lack of a systematic and complete taxonomy of these methods, which should be based on accurate understanding of MM. The various complex processes of gathering, storing, purifying, and circulating Qi should be understood, operationalized, and brought out of the realm of apparent superstition. We do not mean to suggest that the traditional concepts of Qi should be imported into the realm of science; this cannot and should not be done. But both the subjective and the objective phenomena associated with descriptions of Qi can be identified in a scientific way.

We note in the reviews cited above the lack of complete agreement as to the requirements of adequate study design. Whereas some apply the rigorous CONSORT (109) standards in evaluating the acceptability of studies, others suggest more moderate standards such as the Boutron modifications (110) or the Jadad criteria (112), or standards specifically designed for this particular field such as the Wayne checklist (103). Some consensus needs to be reached on this issue to further research in this field.

## Possible Mechanisms of the Effects of MM on Affective Disorders

There are two broad categories of mechanisms proposed for the effects of MM: first, well-recognized mechanisms shared by ordinary physical exercise, such as muscular or cardio-vascular loading; and second, complex neurological mechanisms relating either to neuronal plasticity (128–130) or neurohormonal and neuroimmunological modulation (25, 95). We think the latter offers the most productive avenue for future research, and we make several speculations along these lines below.

### Metabolic expenditure

Meditative movement exercises are of mild to moderate intensity, are easily controlled, need no equipment, and little space, can be done indoors in inclement weather, and involve no sudden movements. The level of exertion is not much more than that of a gentle walk, and it may be hard to understand how MM can have such a range of powerful effects. MM involves smooth coordinated movements of all parts of the body, which gently challenge the range of motion of essential joints. In keeping with its philosophy of balance, MM avoids extremes of stretch or exertion and cautions against any feelings of strain or effort (131).

Studies comparing the effects of MM on physical measures such as balance, bone density, HR, heart rate variability (HRV), BP, aerobic capacity, and strength to a no-intervention control generally found significant improvement in these indices (increased balance and bone density, reduced HR and BP, increased HRV, aerobic capacity, and strength) with MM, whereas comparisons to conventional exercise interventions often showed MM to have equally positive effects as conventional exercise. However in the case of the MM intervention, the degree of exertion appeared to be substantially less than that used in the conventional exercise intervention. Jahnke mentions that the relatively mild leg flexion and low intensity movements typical of MM nevertheless produced significant effects on bone density (19, 103). Jahnke speculated that some of the positive physical effects of MM might not be due to the same mechanisms as those of conventional exercise. More precise measurements of the metabolic equivalent (MET) and other objective measures of exertion will be necessary for clear conclusions. Chao has measured the MET of modified Taijiquan to be three METs, a moderately low intensity level (132). MET is a measure of the energy consumption during exercise, expressed as a ratio to a standard level of energy consumption approximately equivalent to that during a resting state. Thus one MET is a resting state, three METs is three times the energy consumption of the resting state. Maximal exertion would be about 23 METs. Some comparisons of MM to conventional exercise demonstrate greater effects on regulating the autonomic nervous system for MM (18), again suggesting a different mechanism from that of conventional exercise.

### Rhythm

The smooth rhythmic motions of MM are usually experienced as quite pleasurable. Shin Lin (102) suggests that moderate rhythmic movement may increase parasympathetic tone, whereas intense exertion causes more sympathetic activation (102). Both in the elderly and in infants, regular rhythmic motion has been shown to be calming (133). In animal behavior, moderate rhythmic motion is very common in grooming behavior, which has been observed to have a calming effect on the animal. Cats have been shown to release elevated levels of serotonin while grooming (134); it is possible something similar happens in humans when activities mimic grooming behaviors.

The speed with which one performs MM is around six times a minute (0.1 Hz); this appears to be a frequency at which the breath and heart beat have the greatest tendency to come into coherence (135); in other words, at this frequency respiratory sinus arrhythmia (RSA) is enhanced. During MM practice there is a feeling of “being in the groove,” of moving in a strong, slow rhythm. When RSA is strongly established, blood circulation becomes more efficient; the heart rate increases during inhalation, as the blood volume moves relatively more into the lungs, and slows during exhalation, as the blood volume moves into the peripheral circulation. This alteration in blood volume may be part of the basis for the suggestion in MM that one “breathes into the arms and legs” – an obvious physical impossibility, but perhaps a good description of the experience of a regular oscillation of blood volume (136). This promotes efficiency in the cardio-respiratory system, and may enhance parasympathetic tone when such tone is low. In states of excess vagal tone and insufficient sympathetic arousal (vaso-vagal syndrome), this rhythmic breathing could bring the ANS back toward balance by elevating sympathetic activity (137) and possibly reducing vagal tone. This restoration of autonomic balance is likely to have a moderating effect on affective symptoms, and might reduce conscious fear and anxiety by reducing the intensity of the somatic markers (the interoceptive awareness of visceral/affective states on which subjective affective experience may be based) (138). This synchronized breathing will affect measures HRV (139); a frequency analysis of HRV will tend to show a strong narrow spike in the high frequency (HF) range, which is regarded as the most reliable indicator of healthy increase in vagal tone (72), and will show reduced power through the rest of the range. This has been referred to as “coherent breathing” (140), and it may be associated with strong positive feelings and a more balanced mental state (135). Interestingly, the one outcome for which there is the greatest evidence for the effectiveness of Qigong is reduced BP; hypertension can be significantly associated with increased anxiety and stress.

### Grounding and posture

Many MM practices involve a regular shifting of the weight form one foot to the other, or a rhythmical activity of the whole body with the weight firmly planted on both slightly bent legs. Great attention is paid to correct postural alignment and to using the body as a whole. Although the degree of exertion is low, the muscles are being used in a well-coordinated and conscious way, possibly re-patterning the motor nervous system. This aspect of the practice is held to enhance “grounding,” which is the subjective feeling and objective state of being more stably connected to the ground. It seems likely that this experience would help increase the sense of self-efficacy, balance, and confidence, all of which have been shown to be improved by MM (23, 98), and might help to stabilize mood swings and reduce depression and anxiety.

A recent study of posture (141) showed that when subjects adopted a “low-power” or a “high-power” posture for a few minutes, there were significant shifts in behavior as well as in testosterone and cortisol levels. The authors stated that their results indicate the reality of “cognitive embodiment,” in which voluntary physical posture can produce not only changes in cognitive and affective experience, but neurohormonal shifts as well. This suggests that the emphasis on posture in MM may have a scientific basis (142). Carney et al. used typical postures of the kind one might see in a boardroom; from the viewpoint of MM, the postures were unbalanced and poorly integrated (although they do clearly express power or lack thereof). The neurochemical and behavioral shifts that occurred through adopting a “high-power” posture involved increased testosterone and a shift toward risk-taking behavior; this is no more a shift toward balance than the increased cortisol observed in the “low-power” postures. The very precise posture used in almost all MM movements is well aligned and balanced between the extremes. We speculate that just the act of holding a balanced posture for a period of time may induce behavioral, affective, and neurochemical shifts in the direction of balance. A study by Yeh et al. (143) demonstrated a significant decrease in leukopenia in breast cancer patients using a 3-week Standing Meditation Qigong intervention (Zhan Zhuang); this practice involved simply standing for 15 min in a precisely aligned, relaxed, and well-balanced posture. In seated meditation too, the correct posture is seen as a central part of the practice (144).

### Interoception

A central aspect of MM is the attention to interoceptive and proprioceptive sensations. MM practitioners become significantly more sensitive to tactile (129), interoceptive (128), and kinesthetic perceptions, and regularly experience a variety of positive and complex inner sensations. Damasio’s recent theory of somatic markers (145) has clarified how important interoception is to affective and cognitive function; he has shown that what are called intuitions or hunches may be due to this system. Information about the state of the viscera comes up lamina 1 of the spinal cord, via the brainstem parabrachial nucleus to the ventromedial thalamus, and thence through the posterior insular cortex (the primary interoceptive cortex) to the anterior insula where the information is integrated with higher order contextual information and becomes accessible to consciousness. Damasio suggests that the integrity of this pathway is necessary for many affective, interpersonal, and cognitive processes, since it is the basis for a clear sense of one’s own affective and autonomic state (146). Information received through this interoceptive system is the basis on which the anterior insula (as well as the anterior cingulate gyrus and the ventromedial prefrontal cortex) generate an attentional bias, which organizes the brain to pay attention to the outer world in particular ways. This system is implicated in the capacity for empathy and compassion, as well as addiction, anxiety, and depression. Farb (147) found that improved conscious access to the interoceptive cues associated with sadness was associated with decreased depressive symptoms. Meditation and MM have been shown to enhance interoception, even to the point of altering the neuronal connections between the posterior and anterior insula, the crucial bridge through which interoceptive sensations reach consciousness (128).We speculate that positive affective changes from MM may be due in part to this process of enhanced interoception.

### Imagery

Qigong uses phrases such as “direct the Qi,” “gather the Qi,” “move the Qi,” which may be problematic to understand from a biomedical point of view. This capacity is a principal goal of Qigong (148) and other forms of MM (where the term “Energy” is often used instead of “Qi”). A useful way of understanding these terms is to think of “Qi” as a dynamic interoceptive/proprioceptive/kinesthetic/tactile sensation of tingling, vibration, warmth, pressure, or flow. Thus “moving the Qi” translates as the intentional use of imagery to modify the practitioner’s inner experience. A typical MM instruction might be: “While you are performing a certain coordinated movement, imagine the sensation of a flow of warm liquid from your pelvis up through your spine and out your arm.” Or: “place your attention in the center of your chest and imagine a flower gently opening.” After a period of practice, such procedures can result in vivid physical sensations; not an abstract mental picture, but an embodied “felt sense” of interoceptive/kinesthetic experience, usually with a clear hedonic component. These subjectively experienced interoceptive shifts are believed to correspond to actual changes in the physiology of the body and nervous system (58, 131). For instance a spread of pleasant warmth may indicate increased capillary dilation; a soft feeling in the heart may involve reduced BP and increased HRV; a feeling of weightlessness in the limbs may indicate improved control of muscle tone via the reticular activating system in the brain stem.

This process is similar to an athlete’s use of motor imagery for the rehearsal of a sport (149). Visual, interoceptive, and kinesthetic imagery can produce significant, widespread, and lasting changes in the brain. Motor imagery is known to activate areas of the brain responsible for generating internal sensations, such as the posterior parietal cortex and the pre-motor and supplementary motor areas (150–152). Visualization can improve motor performance significantly (149, 153), and increase muscle strength (154). Imagined movement activates many of the same areas of the brain as actual motion, although the patterns of activation are not identical (155). The posterior parietal cortex, where the body image is constructed (156), as well as the supplementary motor area, where motor plans are elaborated, are preferentially activated by imagined movement (151, 157). The effects of imagined movement extend to the autonomic system; cardio-vascular and skin resistance changes accompany imagined exertion (150, 158), and it is possible that other autonomic changes can be triggered as well, such as altered parasympathetic regulation of the heart (140), improved autonomic regulation of the enteric nervous system, and increased capacity for social engagement through activation of the supra-diaphragmatic portion of the parasympathetic system – Porges’ ventro-vagal system (159). The positive effects of motor imagery can be predicted from the degree of autonomic responsiveness during the imagery (160).

### The basal ganglia

The principle functional circuits of the brain: affective, interoceptive, motivational, attentional, executive, associative, memory, and sensorimotor all need to coordinate with each other in relation to the challenges from the environment. One needs to be able to notice and orient to a relevant stimulus, identify its meaning, adopt an appropriate physical, affective, and cognitive preparatory stance (posture), then choose and execute appropriate action. Recent research into the basal ganglia (BG) suggests that they are a major center for this functional integration. All cortical regions have semi-independent parallel loop circuits going down to the striatum (the input nucleus of the BG), to the globus pallidus, substantia nigra, and sub-thalamic nucleus, and returning via the thalamus to the same area of the cortex (161). This enables the BG to selectively inhibit or stimulate particular parts of the cortex, as well as to coordinate their actions. This applies to both voluntary and involuntary or habitual actions (162). The BG have a similar set of loop circuits descending to the pontine and medullary areas, extending the process of selection and coordination to brainstem functions (163). The symptoms of Parkinson’s disease (PD; which is caused by a deficit in the dopamine circuits of the BG), as well as other diseases possibly related to the BG, such as Tourette’s syndrome and attention deficit hyperactivity disorder (ADHD), have associated behavioral, cognitive, and affective symptoms (161). PD is known for restrained movement, contracted posture, depression, and cognitive limitations; ADHD presents an opposite picture. The terms hypophrenia and hyperphrenia have been coined to refer to the cognitive/affective dimensions of hypo- and hyper-kinetic disorders, indicating the interaction of motor, affective, and cognitive dimensions in the BG (161).

We speculate that the focus of MM on the intentional cultivation of balanced posture, enhanced interoception, and kinesthesis, the conscious focus on smooth and balanced movement, rhythmic breathing and positive affect, and the explicit use of intention, all acting together, may affect the whole brain via the BG and move it toward more balanced functioning. Hyperphrenia (anxiety, ADHD) calms down and hypophrenia (depression, apathy) is energized. Excess activation in the HPA axis diminishes, and the neurochemical environment becomes well regulated. In this process posture and movement play a central and fundamental role; the process of “adopting a posture toward something” (164) is a central and unifying function of the entire nervous system. Llinas (165) has argued that motor function is the principle reason for the existence of the CNS.

### Dual default modes of the brain

Recent research has identified rumination or mind-wandering (the chronic semi-conscious churning of thoughts) (166) as a significant factor in anxiety and depression (167–169). The “default mode” of the brain (50, 166) involves activity of the posterior cingulate cortex and the precuneus, as well as the superior and medial temporal gyri; these are both involved with internal “self-talk,” the “autobiographical” self with all its stressful stories (170). Brewer (166) has shown in a study of experienced meditators that in meditative states these regions become less active, and the dorso-lateral and medial prefrontal cortex, the dorsal anterior cingulate cortex, and posterior insular cortex become more active. These are areas involved with cognitive control and attention to the present, particularly to interoceptive stimuli. Long-term meditation practice brings about permanent restructuring of these pats of the brain, resulting for instance in increased gray matter in areas of the cortex involved in interoception (171, 172). We suggest that MM may be effective in the short term at changing the default mode of brain activity away from rumination toward present-oriented awareness, and in the long term at changing the wiring of the brain (128). If this is the case, MM could be of immense help in the therapy of depression and anxiety.

## Pitfalls in Studying MM and Suggestions for Future Research

We have emphasized above the importance of an adequate understanding of the core principles of MM, as well as the development of a systematic taxonomy. We believe this is necessary for significant progress. Research should be carried out in populations with clinical diagnoses of depression or anxiety; however when dealing with clinical illness, an MM intervention should be matched to the condition it is designed to treat. This requires the help of an appropriately qualified practitioner. A confounding factor could be that the diagnosis of the patients in the research group from an MM point of view might not be congruent with that of Western medicine.

### Research design

As noted in Section “Reviews of MM’s Effects on Affective States” above, consensus needs to be reached as to the appropriate standards for conducting and evaluating studies of MM. Recommendations for the design of future research include: adequate sample size, the correct use of blinding procedures, ideally use of a double blind; the use of control groups, ideally three-arm studies using an inactive and an active attention, treatment, or sham intervention control. In reporting outcomes blinding and randomized selection procedures should be fully described. The great majority of studies of the effect of MM on depression or anxiety are limited to about 3 months of intervention. In many cases, this may not be enough time to even begin to achieve mastery; one could say that the real “dose” does not begin until there is a reasonable degree of skill in performing the practices. Trials should be long enough to ensure subjects achieve an adequate level of skill. Skill levels attained by subjects should be measured and taken into account in analysis of the outcomes. We suggest defining certain reported subjective experiences – such as sensations of lightness, flow, warmth, wholeness – as indices of skill levels. Traditional Qigong teachings describe the experiences indicative of various degrees of achievement (113). Objective measures could also be made that might correlate with skill, such as analysis of postural sway or stride variation (173). Measures based on the theory of complex systems could prove productive (111, 173–176).

Since MM uses many variables in intervention, studies need detailed descriptions not only of physical movements, but also of the nature of instructions in terms of use of mind as well as the social context. One shortcoming for all current research is the lack of some sort of taxonomy to break down elements into recognizable categories; until such a system is developed and adopted, it will remain difficult to compare studies and gain significant understanding of MM and disciplines like Qigong.

### Future directions

As the above factors are implemented, clear and solid results should begin to accumulate as to the effects of MM practices. Should these results confirm the suggestions from current research, then it will be appropriate to begin to deconstruct the role of the different aspects of MM: what proportion of the results are due to the movement, the breathing, the mental focus; how much is due to social interaction, positive expectation, cultural belief, and so on. Specific elements of MM should be used in isolation to examine the exact mechanisms of action, and in combination to determine whether the components have an additive or a synergistic effect. As the definition of MM becomes clearly established, more forms of MM should be studied in depth, including specific forms of Yoga and Western Somatics. Care should be taken to discriminate between MM and other factors such as religious belief, devotional practices, and philosophical or moral practices. As more forms of MM are studied the definition of MM should be evaluated and refined to make sure that it remains valid and useful.

## Conclusion

Meditative movement is a system of considerable scope, sophistication, complexity, and potential power. At this early stage it is necessary to consider, with appropriate discrimination, a wide range of explanatory mechanisms of its effects, from ordinary physiological and psychological processes, through higher brain functions, to electrical, and electromagnetic field phenomena.

Meditative movement uses techniques that are quite different from those of conventional exercise, and there are sound possible explanations for their mechanisms of action on affective and autonomic states. Investigation into these putative mechanisms could lead to significant breakthroughs in the development of new forms of intervention for anxiety, depression, and related conditions. We have shown that culturally unfamiliar explanations of the therapeutic effects of MM (such as “moving the Qi”) can be translated into scientifically meaningful terms, allowing for a significantly more thorough and effective investigation of MM disciplines than has been possible hitherto. In particular we believe that is necessary to consider actions via the higher centers of the brain when studying MM.

Given the lack of adverse outcomes in studies that employed various forms of MM, the low costs involved in delivering MM therapies and that the outcomes of most studies are encouraging, we believe that continued research in this area could prove to be highly beneficial in the field of anxiety and depression disorder treatment.

## Conflict of Interest Statement

The authors declare that the research was conducted in the absence of any commercial or financial relationships that could be construed as a potential conflict of interest.
